# Infantile Diseases

**Published:** 1875-06

**Authors:** 


### INFANTILE DISEASES.

We have a letter from an Iowa subscriber who asks us if we encourage patent nostrums,—referring to the "Van Deren Remedy." We reply that we do not, and that we put no faith in cure-alls. We know that this prescription for bowel-diseases of children is the work of an educated and experienced physician, and that it has, in our own practice, accomplished great good. It differs only from other prescriptions, in being put up for the people at large, instead of for one patient only.

